# Exploring the Potential of Pure Germanium Kesterite for a 2T Kesterite/Silicon Tandem Solar Cell: A Simulation Study

**DOI:** 10.3390/ma16186107

**Published:** 2023-09-07

**Authors:** Matas Rudzikas, Saulius Pakalka, Jolanta Donėlienė, Arūnas Šetkus

**Affiliations:** 1 Center for Physical Sciences and Technology, Saulėtekio Av. 3, LT-10257 Vilnius, Lithuania; arunas.setkus@ftmc.lt; 2 The Applied Research Institute for Prospective Technologies, Vismaliukų Str. 34, LT-10243 Vilnius, Lithuania; s.pakalka@protechnology.lt (S.P.); jolanta.doneliene@protechnology.lt (J.D.); 3 Modern E-Technologies, Vismaliukų Str. 34, LT-10243 Vilnius, Lithuania

**Keywords:** kesterite, 2T tandem solar cells, simulations, transfer matrix, SCAPS

## Abstract

Recently, the development of tandem devices has become one of the main strategies for further improving the efficiency of photovoltaic modules. In this regard, combining well-established Si technology with thin film technology is one of the most promising approaches. However, this imposes several limitations on such thin film technology, such as low prices, the absence of scarce or toxic elements, the possibility to tune optical properties and long lifetime stability. Therefore, to show the potential of kesterite/silicon tandems, in this work, a 2 terminal (2T) structure using pure germanium kesterite was simulated with combined SCAPS and transfer matrix methods. To explore the impact of individual modifications, a stepwise approach was adopted to improve the kesterite. For the bottom sub cell, a state-of-the-art silicon PERC cell was used with an efficiency of 24%. As a final result, 19.56% efficiency was obtained for the standalone top kesterite solar cell and 28.6% for the tandem device, exceeding standalone silicon efficiency by 4.6% and justifying a new method for improvement. The improvement observed could be attributed primarily to the enhanced effective lifetime, optimized base doping, and mitigated recombination at both the back and top layers of the CZGSSe absorber. Finally, colorimetric analysis showed that color purity for such tandem structure was low, and hues were limited to the predominant colors, which were reddish, yellowish, and purple in an anti-reflective coating (ARC) thickness range of 20–300 nm. The sensitivity of color variation for the whole ARC thickness range to electrical parameters was minimal: efficiency was obtained ranging from 28.05% to 28.63%.

## 1. Introduction

In order to effectively mitigate climate change, it is crucial to accelerate the use of renewable energy sources [1]. Among them, solar energy stands out as one of the fastest-growing options and holds great potential to become a primary energy source in the future. However, silicon technology, which has the highest market share, is almost at its efficiency threshold. In fact, large-scale commercial solar cells have already achieved efficiencies within the range of 23–26% while their practical limit is just above 27%, considering factors such as extrinsic recombination loss, optical loss, and resistive loss [2]. Consequently, there is an urgent need for the development of tandem solar cells that are both efficient and affordable.

There is a significant trend toward utilizing perovskites in combination with silicon, as they demonstrate remarkable efficiencies. For instance, small-sized cells achieved an impressive efficiency of 31.3% [3], as documented in M. Green Efficiency Tables 61 [4]. Moreover, Oxford PV recently announced an efficiency of approximately 27% for an M6 wafer size [5]. Nonetheless, despite the extensive investment and research, perovskite technology still faces challenges relating to its stability [6].

Another promising technology is based on the kieserite family of compounds. These materials possess noteworthy advantages, as they exhibit excellent stability and do not rely on critical raw materials (CRM) classified by the European Commission [7]. Furthermore, their band gap can be precisely adjusted by alloying with anionic substitutions of Se or S or cationic substitutions of Si, Ge, and Sn within a range spanning from 0.88 to 2.22 eV [8]. However, it is worth noting that despite these advantages, the highest reported efficiency achieved thus far remains at only 14.1% [9], and during the latest 5–8 years, improvements in efficiency were minor [10]. Importantly, the highest obtained efficiencies were for the lower band gap kesterites 1.1–1.2 eV [11]. Nevertheless, the latest efficiency jump published over the last year still keeps kesterite technology as one of the most promising low-cost, high-lifetime thin film technologies with the possibility to be applied both as standalone devices as well as a top sub cell in tandem devices.

Simulations are often used to predict outcomes and improve devices more efficiently, implementing only successful modifications into real devices [12]. A. Jimenez-Arguijo et al. [13] modeled 4 terminal (4T) tandems with kesterite in two cases: (1) as a top sub cell (Cu_2_ZnSnS_4_ or Cu_2_Zn(Sn,Ge)S_4_) with silicon as the bottom sub cell (22.9%) and (2) as a bottom sub cell with perovskite as the top sub cell using SCAPS and transfer matrix methods. It was shown that in the first case, with minor improvements, 30% efficiencies could be reached. In the second case, the authors stated that it was quite ambitious and without any breakthrough: this combination was not a viable option. S. Khelifi demonstrated the enhancement possibilities for pure germanium (Cu_2_ZnGeSe_4_ or CZGSe) kesterite via simulations and then modeled 4T tandem with silicon using SCAPS for electrical calculations and the Lambertian scattering scheme for light trapping [14]. Researchers have shown that kesterite top cells with a minimum efficiency of 14%, a bandgap of 1.5–1.7 eV, and transparency higher than 80% are needed in order to obtain tandem efficiency higher than a single junction silicon cell (25% in the particular work). Some authors also simulated 2T structures. N. Doumit used a hybrid buffer layer of In_2_O_3_/CdS, optimizing its position, thickness, and the doping of the Cu_2_ZnSnS_4_ (CZTS) sub cell for 2T tandem with silicon using COMSOL Multiphysics software. They obtained an efficiency increase from 13.5% to 28.5%. While V. Sivathanu [15] simulated the monolithic CZTS/Si tandem using heterojunction silicon with an efficiency of 14.46% from their previous work [16] using ATLAS 2D simulator, they improved both sub cells achieving a maximum efficiency of 20.17%. Mora-Herrera et al. simulated kesterite solar cell enhancement without tandem integration. Scientists have shown that a particular chalcogenide cell could be improved from 8.3 to 20.6% using a rather complicated kesterite multi-defected absorber in SCAPS [12]. However, most of these works used a non-state-of-the-art silicon sub cell or employed CZTS with a maximum bandgap limitation of 1.6 eV [11], therefore not fully implementing the optical part of simulations. 

Therefore, the aim of this study was to explore the potential of a kesterite/silicon 2T tandem as a future PV technology to replace single junction silicon using simulations. SCAPS was employed to calculate the electrical part and the transfer matrix method (TMM) was used for the optical part. TMM not only served to calculate the optics required as a filter for SCAPS (as in the previous works) but also to fully exclude the parasitic absorption by calculation of carrier generation. Kesterite refractive index spectra were incorporated with a band gap from 1.52 to 2.02 eV via sulfur/selenium alloying (Cu2ZnGe(SxSe1−x)4 or CZGSSe) [17] to determine the optimal kesterite thickness and a bandgap, what was absent or not fully realized in prior research. In total, 24% of state-of-the-art PERC silicon solar cells were used as a bottom device [4]. To explore the impact of individual modifications, a stepwise approach was adopted to improve the kesterite structure. Moreover, the quality of the kesterite absorber was assessed solely through an effective carrier lifetime in the bulk, representing the complex crystal quality of kesterite through a single parameter. This approach is supported by T. Unold’s findings, which demonstrate a strong correlation between this parameter and the record efficiencies achieved in thin film solar cells [18]. The overarching objective is to derive general conclusions regarding the necessary improvements in crystalline quality to establish a viable 2T tandem configuration. Finally, the evaluation of colorimetric properties for the tandem device by varying anti-reflective coating (ARC) thickness is also included in this work, taking into consideration that colored PV panels are beginning to be favored for building integrated photovoltaics (BIPV) [19]. The BIPV market is currently growing due to such advantages as having an energy source closer to the user, not needing an additional surface area, and implementing (nearly) zero-energy buildings.

## 2. Simulation Model

The electrical part of the solar cell was simulated by the SCAPS (version 3.3.10)—a 1D solar cell simulation software, which was developed at the Department of Electronics and Information Systems (ELIS) of the University of Gent, Ghent, Belgium [20].

The Abeles transfer matrix method (TMM) was employed to perform optical simulations on a multilayered structure. The transfer matrix method utilizes 2 × 2 matrices to represent the electromagnetic wave amplitude and phase characteristics of each layer and the interface within the structure. To facilitate the implementation of this method, a Python package developed by Steven J. Byrnes was used [21]. 

The simulation is performed in a four-step procedure for each tandem structure:

1. The SCAPS script generates a mesh file for TMM in both sub cells;
2. The TMM script first calculates reflectance and absorbance in each of the layers of the multilayered structure and then calculates the carrier generation profile for each layer of sub cells;
3. The SCAPS script for electrical calculations of both sub cells generates J–V curves for both sub cells;
4. The Python script adds 2 J–V curves and calculates characteristic parameters (eff, J_SC –_ short circuit current density, etc.).

Assumptions used in the simulations:

- No texture is present, and layers are flat;
- Light is perpendicular to the surface;
- Each layer’s properties are homogeneous inside and change stepwise when moving from one layer to another;
- Carrier generation is calculated using Equation (A1) with exponential decay. However, in reality, light is transmitted and reflected multiple times in a multilayered structure; therefore, full absorption can only be ensured using the normalization of the carrier generation profile, which is integral to the absorption integral. This assumption is thought to have a minimal impact on the results;
- Tandem J–V is obtained by adding J–V curves to both sub cells. However, in most cases, this results in an incomplete J–V curve with the missing low voltages from J_SC_ and up, depending on the current mismatch level (except the current matching conditions). To obtain the full J–V curve, missing points of tandem J–V can be obtained by the linear approximation of 3% of the lowest data points;
- J_SC_ loss is calculated from Equation (A4) and Appendix A assuming an ideal EQE because of the limitation in SCAPS (SCAPS does not provide EQE: when the carrier generation profile is imported, it is not generated inside the software);
- Thick layers like glass, EVA, and silicon are treated as incoherent (the phase is dropped out) in TMM calculations as the coherence length is usually less than 30 µm in films [22];
- Flat bands are assumed in SCAPS at interfaces with contacts;
- One exception was made for the silicon REF cell. The calculation of a carrier generation profile was performed using Equation (A4) from Appendix A. Absorbance was obtained: $A\left(\lambda \right)=1-R\left(\lambda \right)$, where reflectance was taken from Maryam Valiei et al. [23]. This was the case, as silicon cells are already very well developed in the industry, and it is not possible to obtain good light trapping without the presence of the texture and precise replication of the cell’s structure;
- Only the SRH recombination was considered in this work (Auger and band-to-band were not);
- In addition, it should be noted that alternatively, MoO_3_ can be used as a tunnel junction for such a tandem device that also creates a blocking layer for atom diffusion during the sulfurization/selenization at 450–550 °C of the top sub cell. However, due to the limitations in SCAPS with a maximum of seven layers, this was not realized. Therefore, an ideal junction was considered in this work. However, this is a minor point, not interfering with the main message of the work.

## 3. Simulation Data

### 3.1. Data Used for the Simulations to Describe Materials

The main material properties for the simulation were taken from the relevant literature and are listed in Table 1.

**Figure 1:**
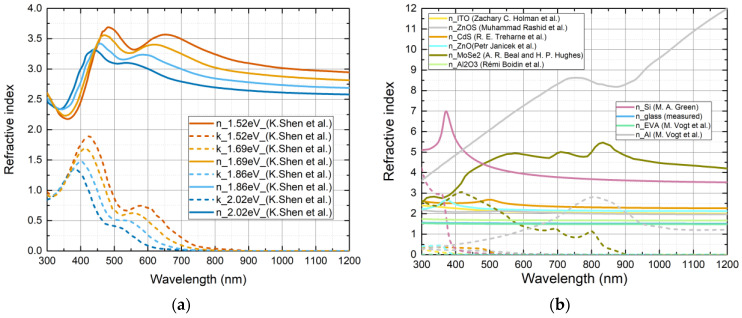
Refractive indexes of Cu_2_ZnGe(S_x_Se_1−x_)_4_ solid solutions with different anion ratios (**a**) [17] and other layers that were used in simulations (**b**): ITO (yellow) (n conc: 2 × 10^20^ cm^−3^) [27], ZnO_0.25_S_0.75_ (light grey) [28], CdS (orange) [29], ZnO (clear sky blue) [30], MoSe_2_ (brown) [31], Si (purple) [32], glass (blue) (measured), EVA (green) [33], Al_2_O_3_ (aquamarine) [34], Al grey) [35].

Refractive indexes for CZGSSe were taken from theoretical calculations (DFT) from Kesheng Shen et al. [17] and are depicted in Figure 1a. The refractive index data of other layers are shown in Figure 1b. 

### 3.2. Reference Sub Cells for the CZG(S,Se)/Silicon Tandem Device

At the initial stage, the selection of the kesterite REF device was based on two main criteria: the highest efficiency achieved by previously reported pure germanium kesterite devices in the literature and the band gap compatibility with available refractive index spectra data (CZGSe E_g_ = 1.45 eV). Consequently, a solar cell from I. Anefnaf et al. [36], with an efficiency of 6.5% and a band gap of 1.52 eV, was chosen for this study.

**Figure 2:**
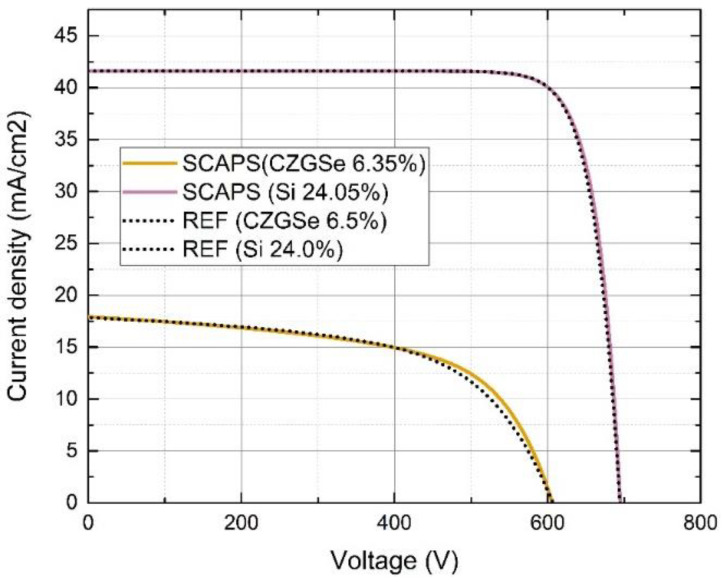
Comparison of REF cell IV curve to the SCAPS cells.

To accurately mimic the J–V characteristics of the real cell using the virtual SCAPS device, the parameters of the kesterite cell were adjusted to obtain the best fit (Figure 2). These parameters included the CZGSe acceptor’s concentration (Na) and the total trap density (N_t_), as well as the MoSe_2_ acceptor concentration and total trap density. The best fit was obtained with the following values: N_a_ (CZGSe) = 10^14^ cm^−3^, N_t_ (CZGSe) = 3.3 × 10^17^ cm^−3^, Na (MoSe_2_) = 2 × 10^15^ cm^−3^, N_t_ (MoSe_2_) = 10^18^ cm^−3^, resulting in the effective lifetime of the kesterite absorber (τ) of 1.1 × 10^−11^ s. Additional parameters were assigned the following values: the interface recombination velocity between the kesterite absorber and the buffer layer (CdS) was set to 10^5^ cm/s, and electron and hole capture cross-sections—10^−14^ cm^2^. The energy level with respect to the reference was set at 0.6, and the defect type was considered neutral. These parameters were then set as initial ones for the kesterite solar cell and were not modified (if not specified) at any optimization step presented below.

For the silicon REF device, one of the top-performing high-area solar cells with a 24.0% efficiency was selected, manufactured by LONGi [4]. 

The parameters of both real and SCAPS devices are listed in Table 2.

**Figure 3:**
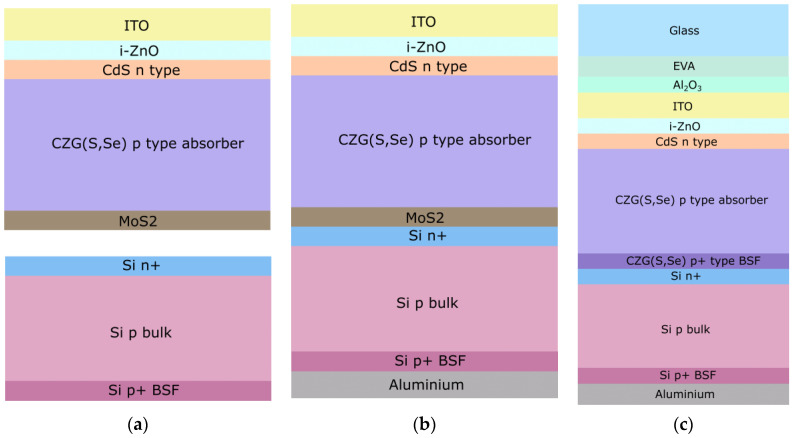
Solar cell structures used in research: baseline REF cells (**a**), baseline 2T tandem (**b**) and optimized 2T tandem, and the optimized 2T tandem structure (**c**).

The structure of the REF cells (a, b) and the optimized 2T tandem structure (c) are depicted in Figure 3. The layered arrangement of the kesterite REF cell was as follows (from top to bottom): ITO, intrinsic ZnO, n-type CdS buffer layer, p-type CZGSe absorber, and MoSe_2_. By contrast, a much simpler solar cell structure was employed for the silicon cell, consisting of n+, p-type bulk, and p^+^ back surface field (BSF). Both cells were simplified to be described by a single parameter, namely, the effective lifetime of the minority charge carrier, described in Appendix B (for the kesterite sub cell, the interface between CdS and CZGSe or CZGSSe was also taken into account). 

## 4. Results: Simulation Results Using Theoretical Refractive Index Data for Kesterite

The following steps were selected for the optimization of the kesterite/silicon tandem device:

- Encapsulation added (2 mm glass + 470 μm EVA);
- MoSe_2_ was removed to reduce parasitic absorption (due to the low bandgap of MoSe_2_) and the p^+^ back surface field (BSF) was added to the kesterite absorber;
- CdS was changed by ZnOS to reduce parasitic light absorption due to the low bandgap of CdS;
- CZGSe’s base doping increased from 10^14^ to 10^16^, according to S. Khelifi [14], and interface recombination velocity (at CdS/CZGSe interface) decreased from 10^5^ to 10^3^ cm/s;
- CZGSe’s effective carrier lifetime varied to observe its effect, and the requirements for obtaining an efficient tandem device;
- CZGSe thickness and band gap (by partial substitution of Se with S) were varied in order to see its effect and the requirements for obtaining an efficient tandem device;
- An Al_2_O_3_ anti-reflective coating was added, and its thickness was optimized. The color effect on current matching and efficiency on the tandem device was observed.

Detailed explanations are provided in the following sections. The results of stepwise optimization are summarized in Table 3 and Table A1 (Appendix C), where the main electrical characteristics and optical loss are listed, respectively.

### 4.1. REF Cells Connected in Series and Encapsulation Added

In the initial stage, REF cells were simply connected in series to form the tandem device without any other modifications. This resulted in the PV device exhibiting poor performance, achieving only 10.21% efficiency. The silicon sub cell, after undergoing light filtering, retained a mere 5.14% efficiency, while the kesterite sub cell demonstrated a similar efficiency to the REF device, standing at 6.17%. Importantly, there was a high current mismatch between the two sub cells: 17.56 vs. 9.7 mA/cm^2^.

**Figure 4:**
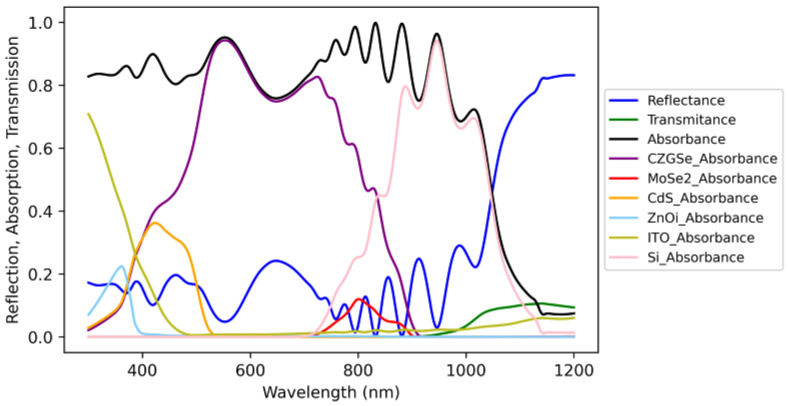
Absorbance in each of the layers of a tandem cell calculated by the transfer matrix method (REF cells connected in series).

The highest optical loss was attributed to reflectance with an integrated current of 10.29 mA/cm^2^ (Table A1 from Appendix C). Upon examining the spectral distribution depicted in Figure 4, it became apparent that a major portion of reflectance occurred in the infrared (IR) range, where it could potentially be absorbed by the silicon sub cell. This occurrence was likely due to the absence of texture. Furthermore, two other notable layers that contributed to parasitic absorbance were ITO and CdS, with values of 1.59 and 1.92 mA/cm^2^, respectively.

**Figure A1:**
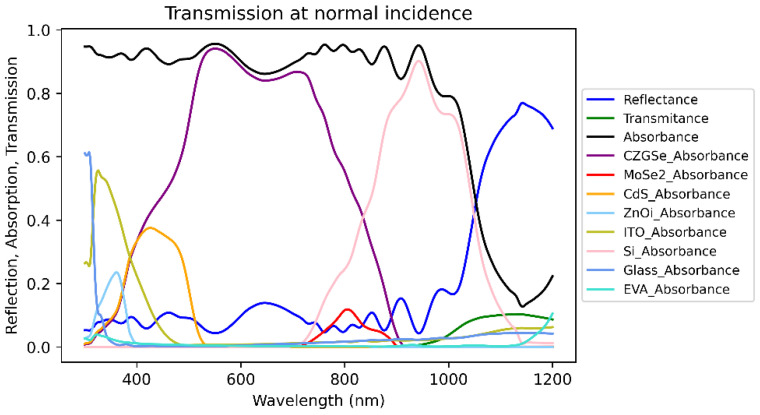
Absorbance in each of the layers of a tandem cell was calculated by the transfer matrix method (after the 2nd optimization step and the addition of encapsulation).

As optical simulations and optical optimization are a significant part of this research, adding encapsulation layers is a necessary step, which is often omitted from the research on solar cells. For this, 2 mm thick glass and 470 μm EVA were added to the setup. Such a modification led to a notable reduction in the current density loss from reflectance, decreasing it from 10.29 to 7.86 mA/cm^2^ (Figure A1 and Table A1 from Appendix C). However, this addition also introduced additional parasitic absorption losses: 0.78 mA/cm^2^ for the glass layer and 0.28 mA/cm^2^ for the EVA layer. As a result, the overall efficiency of the tandem device increased only marginally by 0.2%.

### 4.2. Mitigating Parasitic Loss and Carrier Recombination

In this section, some improvements, which are already known from the scientific community of chalcogenide and chalcopyrite researchers, were added to the tandem device to mitigate the following: MoSe_2_ parasitic absorption, carrier recombination at the back of the kesterite absorber, CdS parasitic absorption and CdS/kesterite interface defects, with no optimal kesterite base doping.

As a second step, MoSe_2_ was removed, and 30 μm p^+^ BSF was introduced into the CZGSe absorber layer. MoSe_2_ was anticipated to primarily benefit the silicon cell by enhancing transmission, while the added BSF was aimed to mitigate recombination at the kesterite’s back surface, thereby mostly improving V_OC_ and J_SC_ through decreased carrier recombination and enhanced carrier collection. The optimum conditions were obtained with 30 μm BSF thickness and doping with a linear change from 10^17^ to 10^18^ cm^−3^. 

This adjustment yielded significant improvements in the kesterite sub cell’s performance: V_OC_ increased from 0.608 V to 0.842 V, J_SC_ increased from 18.38 to 19.84 mA/cm^2^, and efficiency rose from 6.46% to 11.44%, while in the silicon cell, J_SC_ slightly increased from 9.86 to 11.12 mA/cm^2^, resulting in a modest efficiency increase from 5.22% to 5.9%. Ultimately, the tandem device achieved an efficiency of 14.6%, representing a substantial improvement compared to the previous figure of 10.41%.

Then, an attempt to mitigate the parasitic absorption of CdS was made by replacing it with ZnO_x_S_1−x_ (x = 0.8), where the integrated current loss was determined to be 2.06 mA/cm^2^ (Table A1, Appendix C). However, the results revealed an unexpected increase in absorption, reaching 2.29 mA/cm^2^ when using the specific ZnO_0.2_S_0.8_ formulation employed in the simulations (represented mainly by refractive index spectra and parameters listed in Table 1). As a consequence, the efficiency of the tandem device experienced a slight decline from 14.6% to 14.46%. Consequently, for subsequent steps, CdS was retained as it outperformed ZnOS. It should be noted that although CdS has its drawbacks, record kesterite devices were obtained with a CdS buffer [10], and, therefore, extensive research is needed for alternative buffer layers and their optimization.

In the fourth step, kesterite base doping was increased from 10^14^ to 10^16^ cm^−3^, according to S. Khelifi [14], and interface recombination velocity decreased from 10^5^ to 10^3^ cm^−3^. The latter improvements increased kesterite FF from 68.6 to 80.61%, with a slight improvement in J_SC_ and V_OC_. Therefore, the kesterite sub cell and tandem device efficiency rise was observed from 10.61 to 13.56% and from 14.46 to 15.09%, respectively. 

### 4.3. CZGSSe Crystal Quality Represented by Effective Carrier Lifetime

As mentioned in the Introduction section, the complex crystal quality of the kesterite absorber was represented solely through an effective carrier lifetime in the bulk. In addition, only SRH recombination was considered in this work (Auger and band-to-band not).

**Figure 5:**
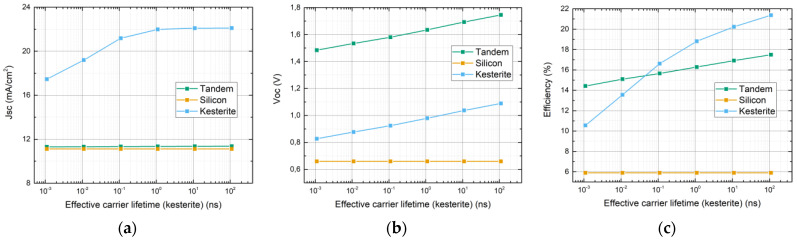
Short circuit current density (**a**), Voc (**b**) and efficiency (**c**) dependence on effective minority carrier lifetime in the kesterite absorber.

The effective minority carrier lifetime systematically varied across a range spanning from 1.1 × 10^−3^ to 1.1 × 10^2^ ns. The corresponding results are presented in Figure 5, revealing a notable trend. It is evident that the open-circuit voltage (V_OC_) exhibits a linear-like increase with the lifetime (a). However, the dependence of J_SC_ from the lifetime shows a saturation effect within the 10–100 ns lifetime range, which was reached between 0.1 and 1 ns, possibly representing carrier collection efficiency improvement (b). In the previous studies, kesterite minority carrier lifetimes have been reported in the range of several ns [39,40,41]. In addition, it is known that layer quality/purity improvement is increasingly higher for higher lifetimes and it can be seen from the figures that higher than 10 ns lifetimes do not provide significant improvements to the cell. Therefore, after careful consideration, an optimal lifetime value of 11 ns was selected to be used in the optimization.

It should be noted that for the selected carrier lifetime of 10 ns, a kesterite sub cell fill factor of 88.34% was obtained. This is quite a high value; however, the purpose of this work was to show the possibilities of the proposed structure using realistic parameters. Considering that the large area (274.1 cm^2^) silicon record solar cell efficiency with an efficiency of 26.6% has a fill factor of 85.6% [4] (made by LONGi), the proposed fill factor is possible but challenging. 

### 4.4. Effect of CZGSSe Thickness and Band Gap

One of the most essential 2T tandem requirements is current matching. In all the preceding steps, current mismatch conditions were observed with a 1.45 μm thick kesterite absorber and a band gap of 1.52 eV. Therefore, at this stage, the thickness and band gap of the kesterite sub cell were systematically varied (along with refractive index spectra and electron affinity) to determine tendencies and to find the optimal conditions for achieving maximum efficiency. Note that the used values of the thickness and the band gap can be easily obtained experimentally. Thus, relatively good control of the layer thickness is feasible for any deposition techniques in the whole range of 0.5–2.0 μm, while an increase in the band gap could be easily achieved by the partial substitution of Se by S, which was previously shown to be beneficial and led to record devices for kesterite technology [9,10].

**Figure 6:**
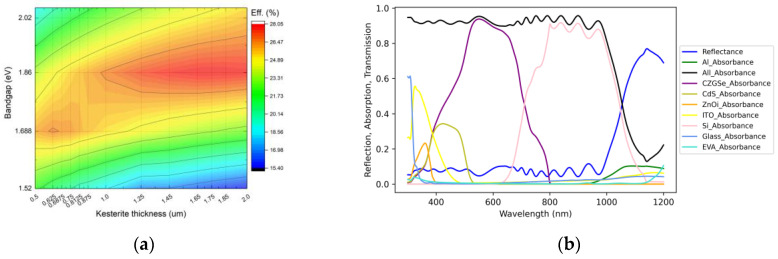
Contour plot of simulated 2T device efficiency from kesterite absorber band gap and thickness (**a**). Absorbance in each of the layers of an optimized tandem cell calculated by the transfer matrix method (**b**).

The results of this optimization process are presented in Figure 6a as a contour plot. Notably, the plot exhibits two prominent maximum points, first located at a thickness of 0.625 μm and a bandgap of 1.688 eV and second at a thickness of 1.65 μm and a bandgap of 1.86 eV. Remarkably, under these optimized conditions, tandem efficiency experienced a substantial increase, reaching 26.58% at the first point and 28.05% at the second. In the latter position, the efficiencies of each of sub cells were as follows: 19.65% for kesterite and 8.72% for silicon.

In addition, it should be noted that the CZGSe spike-like (+0.21 eV) conduction band offset (CBO) was observed with the parameters used for the simulations. It decreased for the CZGSSe (1.688 eV) to +0.042 eV and switched to an electron-favorable cliff-like offset for the higher band gap CZGSSe (1.86 and 2.02 eV), according to the parameters used for the simulations. To conclude, the lower barrier of 1.688 and higher band gap kesterite materials used for the simulations should have contributed to the efficiencies obtained.

Figure 6b presents the absorbance spectra of each layer in the multilayered structure of the tandem cell. It is worth noting that the highest parasitic loss could be primarily attributed to the overall reflectance, as well as the ITO and CdS layers, with corresponding values of 7.29, 1.64, and 2 mA/cm^2^ (respectively).

### 4.5. Al_2_O_3_ Anti-Reflective Coating Added and Its Thickness Optimized

**Figure 7:**
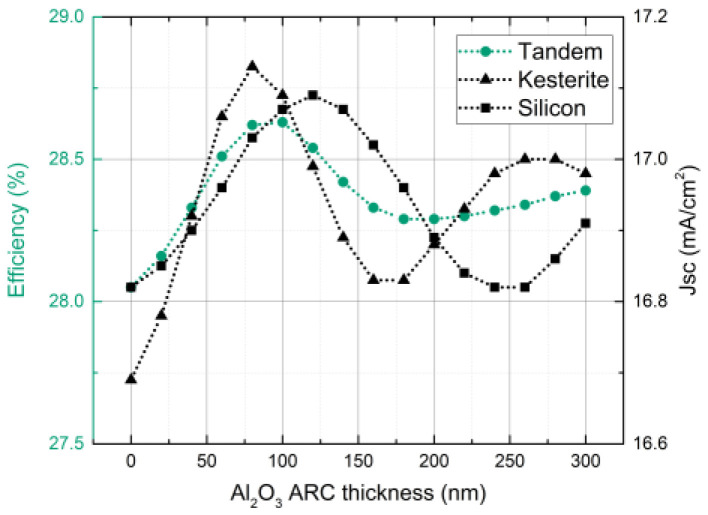
J_SC_ variation in each sub cell and the efficiency of the tandem solar cell using a change in Al_2_O_3_ anti-reflective coating thickness. Efficiency is depicted in the left Y axis, while J_SC_ is in the right.

Al_2_O_3_ ARC (aluminum oxide antireflective coating) was introduced, and its thickness was adjusted for the final optimization step. In addition, the acceptability of this layer for the coloring of tandem cells was tested. The dependence of J_SC_ and its efficiency on the thickness of aluminum oxide is illustrated in Figure 7 (right and left axis, respectively). Notably, the greatest efficiency of 28.63% was achieved with an ARC thickness of 100 nm.

## 5. Colorimetry and Tandem Viability for BIPV

**Figure 8:**
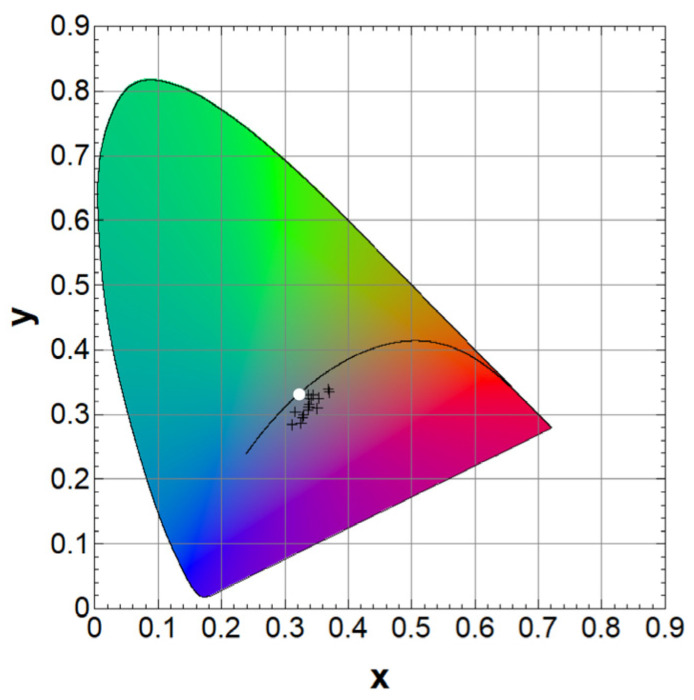
CIE diagram of tandem structures with varying thickness of Al_2_O_3_. It was drawn using software “Benwin+”.

Anti-reflective coating (ARC) with Al_2_O_3_ thickness varying from 20 to 300 nm was added to the top of the structure, as it is shown in the Figure 3c. Reflectance spectra were calculated for various thickness values of an ARC, enabling the evaluation of colorimetric properties of the optimized tandem structure. Characteristic color parameters are displayed in CIE color space in Figure 8. In the figure, the data points are located close to D65 illuminant (represented by the white point), indicating low color purity. The predominant hues are reddish, yellowish, and purple, as most points are clustered in the lower-right part relative to the D65 point.

It can be concluded that the efficiency of the tandem structure exhibits minimal sensitivity to color variations, with an efficiency range of 28.05% to 28.63%. This corresponds to a relative change in efficiency of approximately 2%. However, within this range of the ARC thicknesses, the achievable hues remain limited, resulting in lower color purity compared to the ARC thickness variation with single/double ARC coatings in the literature [42,43,44].

## 6. Discussions

As was mentioned in the Introduction section, silicon solar cells are already very well developed in the industry, with commercial efficiencies ranging from 23 to 26%. However, it was revealed in this study using simulations that silicon cells performed at only 9.11% even at an optimized tandem with 28.6% efficiency (without texture). These findings suggest that the development of silicon cells for tandem applications may not require silicon cells to be this well developed. Instead, it becomes crucial to optimize them for the infrared (IR) region. Doing so could potentially reduce costs without compromising the efficiency of tandem devices.

Another important point for discussion is that material parameter data for simulations are very limited in the literature [18]. It would greatly benefit the research community if more comprehensive data on material parameters, including specific details and processing routes, were measured and shared as a standard practice [18]. For example, experimental refractive index spectra for pure germanium kesterite exist only for ending materials: CZGSe–CZGS [45,46,47], but not intermediate CZGS_x_Se_1−x_. This is very important as, according to the Shockley–Queisser limits, the upper cell needs to have a band gap of 1.73 eV in a 2T configuration with silicon as a bottom sub cell [48]. However, for real devices, certain limitations can occur, for example, on thickness or bandgap, and, therefore, the optimum band gap can shift, as was shown in this work. However, despite limited information on the real parameters of these materials, we believe that the presented optimization steps can be achieved in an experimental way and could lead to a significant improvement in the band gap kesterite-based devices suitable not only for the tandems but also for several applications where semitransparent devices can be used (like BIPV, agrivoltaics, etc.).

## 7. Conclusions

Through a combination of SCAPS electrical calculations and transfer matrix optical calculations, it was shown that the pure germanium kesterite sub cell could be improved from 6.5% with a multi-step process to a reasonable efficiency of 19.56%. This resulted in 28.6% of the tandem device efficiency, which is 4.6% higher compared to the state-of-the-art silicon PERC cell with 24% efficiency used in this work. These values were obtained for 1.65 μm thick and 1.86 eV band gap of the kesterite sub cell. This makes this tandem an attractive option, taking into consideration that texture was absent in the simulations, and reflectance resulted in an integrated current of 6.6 mA/cm^2^, with a considerable part of the IR region. This also suggests that the bottom sub cell could be specifically optimized for the IR part to make it cost-efficient for the implementation of silicon in tandem devices as a bottom sub cell. 

The highest parasitic loss in the final devices was attributed to ITO and CdS, with integrated currents of 1.66 and 1.96 mA/cm^2^. These results indicate potential areas for future improvements.

Moreover, it was obtained that the most significant impact could be attained by increasing the kesterite absorber lifetime from 1.1 × 10^−2^ to 11 ns, which is around one order above the reported lifetime values of experimental works of several ns. This finding holds great importance, considering that enhancing crystalline quality becomes progressively more challenging when aiming for higher minority carrier lifetimes.

Finally, colorimetric analysis revealed that the color purity of this particular tandem structure is relatively low, and hues are limited to the predominant colors, which are reddish, yellowish, and purple in the anti-reflective coating (ARC) thickness range of 20–300 nm. The sensitivity of color variation for the whole ARC thickness range of electrical parameters was minimal: efficiency was obtained, ranging from 28.05% to 28.63%. This suggests that the utilization of this or a comparable tandem device in BIPV could face certain limitations.

## Figures and Tables

**Table 1:** Parameters used for electrical simulation part with SCAPS. References: M. D. Haque et al. [24], S. Khelifi et al. [14], D. Mora-Herrera et al. [12], Muhammad Rashid et al. [25], F. Jafarzadeh et al. [26], A. Jimenez-Arguijo et al. [13].

Parameter	MoSe_2_ [24]	CZGSe [14]	CdS [14]	ZnO_0.2_S_0.8_ [25]	ZnO [14]	ITO [26]	Si (Bulk) [13]
d (μm)	0.02	1.45	0.050	0.050	0.050	0.200	200
E_g_ (eV)	1.29	1.5	2.4	3.07 [12]	3.3	3.65	1.12
χ (eV)	4.2	4.41 **	4.2	4.3 [12]	4.4	4.8	4.5
ε (relative)	13.6	6.950 [12]	8.28 [12]	9	7.8 [12]	8.9	11.9
μ_n_ (cm^2^/V∙s)	100	100	100	100	100	10	150
μ_p_ (cm^2^/V∙s)	25	20	25	25	25	10	45
N_d_ (cm^−3^)	-	-	10^17^	3.93 × 10^18^	10^18^	10^19^	-
N_a_ (cm^−3^)	2.25 × 10^15^ *	10^14^ *	-	-	-		3 × 10^15^
CB effective density of states (1/cm^3^)	2.2 × 10^18^	1.9 × 10^18^	3.1 × 10^18^	2.2 × 10^18^	3.6 × 10^18^	5.2 × 10^18^	2.8 × 10^19^
VB effective density of states (1/cm^3^)	1.8 × 10^19^	1.5 × 10^19^	1.4 × 10^19^	1.8 × 10^19^	1.1 × 10^19^	1.8 × 10^18^	1.04 × 10^19^
electron thermal velocity (cm/s)	2.2 × 10^7^	(2.7 × 10^7^) [12]	(2.3 × 10^7^) [12]	10^7^	(2.2 × 10^7^) [12]	10^7^	10^7^
hole thermal velocity (cm/s)	1.5 × 10^7^	(1.3 × 10^7^) [12]	(1.4 × 10^7^) [12]	10^7^	(1.5 × 10^7^) [12]	10^7^	10^7^

*—set during approximation of reference cell for the IV characteristics. **—for CZGS_x_Se_1−x_ band gap and electron affinities were changed according to the S. Khelifi et al. [14]: 1.52, 1.688, 1.86, 2.05 eV and 4.41, 4.242, 4.074, 3.91 eV (respectivelly). Other parameters were kept the same as for CZGSe.

**Table 2:** Comparison of REF cell parameters to SCAPS cells (simulated as a seperate cells, not in a tandem).

	J_SC_, mA/cm^2^	V_OC_, mV	Efficiency, %	FF, %	τ, s *	Source:
REF CZGSe cell	17.8	606	6.5	60	? **	I. Anefnaf et al. [36]
My REF CZGSe cell SCAPS	17.92	606	6.35	58.51	1.1 × 10^−11^	
REF silicon cell ***	41.58	694	24.0	83.3	? **	M. Green [37]
My REF silicon cell SCAPS	41.50	694.3	23.97	83.2	1 × 10^−3^ ****	

*—Effective minority carrier lifetime at absorber layer. **—Lifetime was not mentioned in these papers. ***—REF silicon cell was taken from M. Green efficiency tables 61 from page 4, Table 2 [4]: “LONGi, p-type PERC”. ****—selected according to silicon lifetimes in the literature [38].

**Table 3:** Main electrical characteristics of stepwise improvement of 2T Si/kesterite tandem device. In step 0, both sub cells were stacked in a tandem configuration without any other modifications.

Step	CZGSSe				Silicon				Tandem			
	V_OC_, V	J_SC_, mA/cm^2^	Eff, %	FF, %	V_OC_, V	J_SC_, mA/cm^2^	Eff, %	FF, %	V_OC_, V	J_SC_, mA/cm^2^	Eff, %	FF, %
0	0.606	17.56	6.17	58.0	0.655	9.7	5.14	80.91	1.259	9.83	10.21	82.54
1	0.608	18.38	6.46	57.8	0.655	9.86	5.22	80.97	1.26	9.99	10.41	82.71
2	0.842	19.74	11.44	68.82	0.659	11.12	5.9	80.62	1.499	11.31	14.6	86.1
3	0.842	18.38	10.61	68.6	0.659	11.07	5.88	80.6	1.497	11.26	14.46	85.77
4	0.877	19.19	13.56	80.61	0.659	11.12	5.9	80.62	1.534	11.32	15.09	86.92
5	1.037	22.09	20.23	88.34	0.659	11.12	5.9	80.62	1.693	11.35	16.91	88.0
6	1.293	16.69	19.09	88.49	0.671	16.82	8.97	79.47	1.959	16.75	28.05	85.47
7	1.293	17.09	19.56	88.54	0.671	17.07	9.11	79.52	1.961	17.32	28.63	84.3

## Data Availability

The data presented in this study are available on request from the corresponding author.

## Appendix A

### Appendix A.1. Calculations of Carrier Generation Profile

The carrier generation profile was calculated using an equation from the book by A. Luque and S. Hegedus [49]:

(A1) $$G(x)=(1-s)\int_{\lambda_{1}}^{\lambda_{2}}A\left(\lambda \right)\Phi_{0}\left(\lambda \right)\alpha \left(\lambda \right)e^{-\alpha x}d\lambda ,$$

where *s*—metal grid coverage ratio (considered 0), $A\left(\lambda \right)$—absorption, $\Phi_{0}\left(\lambda \right)$—photon flux from solar AM1.5g spectrum, $\alpha \left(\lambda \right)$—absorption coefficient, *x*—layer thickness.

Solar spectrum AM1.5g was taken from NREL [50].

The absorption coefficient was calculated from the imaginary part of refractive index spectrum using:

(A2) $$G\alpha \left(\lambda \right)=\frac{2\pi k\left(\lambda \right)}{\lambda }$$

Photon flux was calculated from irradiance:

(A3) $$\Phi_{0}\left(\lambda \right)=I\left(\lambda \right)\frac{\lambda }{hc}$$

### Appendix A.2. Calculation of Maximum Possible J_SC_ and J_SC_ Loss

Maximum possible J_SC_ and J_SC_ losses were calculated according to (EQE is not included as SCAPS does not generate EQE when the carrier generation is imported—not calculated inside):

(A4) $$J_{SC}(loss,gain)=q\int_{\lambda_{1}}^{\lambda_{2}}A\left(\lambda \right)\Phi_{0}\left(\lambda \right)d\lambda$$

Then, each point in the generation profile is multiplied with a constant to make the maximum J_SC_ equal to J_SC_ calculated from the generation profile:

(A5) $$J_{SC}(max)=q\int_{x_{1}}^{x_{2}}G\left(x\right)dx$$

## Appendix B

### Model for Effective Minority Carrier Lifetime and Interface Recombination Velocity

Effective minority carrier lifetime is normally a sum of Auger, Shockley–Read–Hall (SRH) trap-assisted and band-to-band lifetimes:

(A6) $$\frac{1}{\tau_{eff}}=\frac{1}{\tau_{band}}+\frac{1}{\tau_{auger}}+\frac{1}{\tau_{trap}}$$

In this work, only trap-assisted recombination was considered where lifetime was obtained:

(A7) $$\tau_{trap}=\frac{1}{{\sigma N_{t}v}_{th}}$$

Therefore, for the variation in the minority carrier lifetime, the total trap density was varied.

Interface recombination velocity was calculated as follows:

(A8) $$S_{i}=N_{t}\sigma v_{th,}$$

where *N_t_* is the total number of defects, *σ*—electron/hole carrier capture cross sections, *v_th_*—thermal electron/hole velocity.

## Appendix C

### Additional Result Data

**Table A1:** Optical loss calculated for each layer of the 2T tandem structure (in mA/cm^2^).

Step	Reflectance	Glass	EVA	Al_2_O_3_	ITO	ZnO-i	CdS	ZnO_x_S_1−x_	CZGSSe	MoSe_2_	Si	Aluminum
1	10.29	-	-	-	1.59	0.25	1.92	-	21.19	0.71	9.74	0.78
2	7.86	0.78	0.28	-	1.64	0.25	2.06	-	22.21	0.71	9.90	0.78
3	7.47	0.77	0.28	-	1.64	0.25	2.06	-	22.05	-	11.16	0.78
4	8.67	0.78	0.29	-	1.67	0.25	-	2.29	20.64	-	11.11	0.78
5	7.47	0.77	0.28	-	1.64	0.25	2.06	-	22.05	-	11.16	0.78
6	7.47	0.77	0.28	-	1.64	0.25	2.06	-	22.05	-	11.16	0.78
7	7.27	0.77	0.28	-	1.64	0.25	1.93	-	16.70	-	16.84	0.78
8	6.60	0.76	0.28	0.00	1.66	0.25	1.96	-	17.08	-	17.09	0.79
